# Roles of lncRNAs in childhood cancer: Current landscape and future perspectives

**DOI:** 10.3389/fonc.2023.1060107

**Published:** 2023-02-27

**Authors:** Fei Liu, Qian-Wen Xiong, Jin-Hu Wang, Wan-Xin Peng

**Affiliations:** ^1^ Department of Nephrology, Children’s Hospital, National Clinical Research Center for Child Health, Zhejiang University School of Medicine, Hangzhou, China; ^2^ Department of Surgical Oncology, Children’s Hospital, National Clinical Research Center for Child Health, Zhejiang University School of Medicine, Hangzhou, China; ^3^ Cancer Center, Zhejiang University, Hangzhou, China

**Keywords:** childhood cancer, lncRNA, function, biomarker, perspective

## Abstract

According to World Health Organization (WHO), cancer is the leading cause of death for children and adolescents. Leukemias, brain cancers, lymphomas and solid tumors, such as neuroblastoma, ostesarcoma and Wilms tumors are the most common types of childhood cancers. Approximately 400,000 children and adolescents between the ages of 0 and 19 are diagnosed with cancer each year worldwide. The cancer incidence rates have been rising for the past few decades. Generally, the prognosis of childhood cancers is favorable, but the survival rate for many unresectable or recurring cancers is substantially worse. Although random genetic mutations, persistent infections, and environmental factors may serve as contributing factors for many pediatric malignancies, the underlying mechanisms are yet unknown. Long non-coding RNAs (lncRNAs) are a group of transcripts with longer than 200 nucleotides that lack the coding capacity. However, increasing evidence indicates that lncRNAs play vital regulatory roles in cancer initiation and development in both adults and children. In particular, many lncRNAs are stable in cancer patients’ body fluids such as blood and urine, suggesting that they could be used as novel biomarkers. In support of this notion, lncRNAs have been identified in liquid biopsy samples from pediatric cancer patients. In this review, we look at the regulatory functions and underlying processes of lncRNAs in the initiation and progression of children cancer and discuss the potential of lncRNAs as biomarkers for early detection. We hope that this article will help researchers explore lncRNA functions and clinical applications in pediatric cancers.

## Introduction

Childhood malignancies are fundamental diseases of dysregulated development that arise in the context of actively growing tissues. Thus, they often are different from those seen in adults. The most common cancers of children are: leukemia, lymphoma (including both Hodgkin and non-Hodgkin), brain and spinal cord tumors, neuroblastoma other peripheral nervous cell tumors, renal tumors, rhabdomyosarcoma, retinoblastoma, malignant bone tumors (including osteosarcoma and Ewing sarcoma), hepatic tumors, germ cell tumors and additional rare cancers (1, 2). Unlike adult cancers, the overall mutational burden of childhood cancer is low (3). Nevertheless, childhood cancers exhibit genetic, morphological and clinical heterogeneity, which limits the efficacy of existing treatment modalities (4, 5).

**Figure 1:**
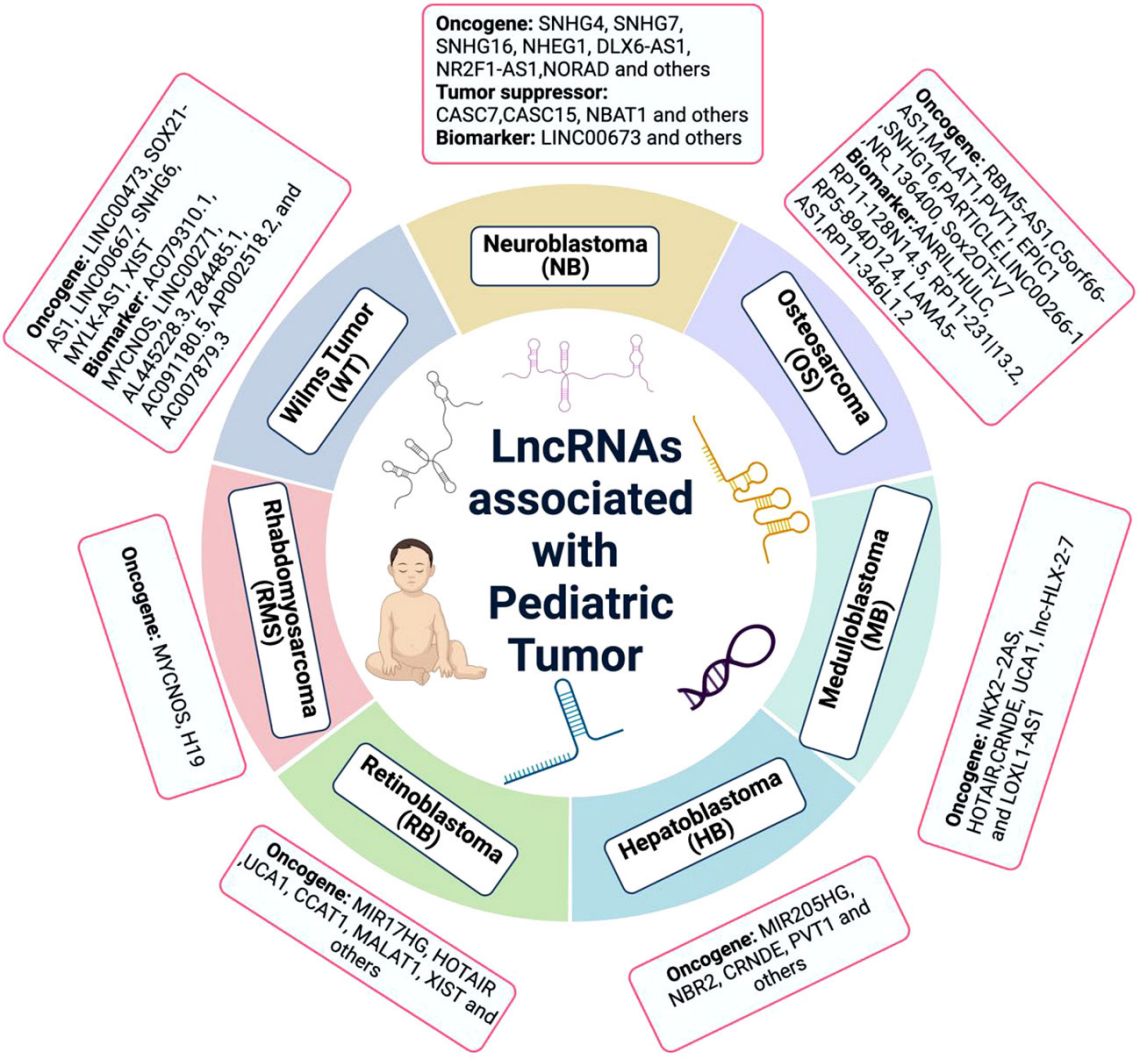
The lncRNA associated with pediatric cancer. Many lncRNAs have been discovered to be dysregulated in pediatric cancers. While, the lack of deep understanding of molecular mechanism is the huge obstacle at present.

For decades, cancer biology focused largely on the protein-encoding genes. It is now well-recognized that only ~2% of the human genome encodes proteins, while the majority of the human genome encodes large numbers of noncoding RNAs (ncRNAs) (6, 7). Among them, the transcripts with a length of more than 200 nucleotides are defined as long non-coding RNAs (lncRNAs) (8), although recent studies demonstrated that some lncRNAs are capable of producing functional small peptides (9, 10). LncRNAs have attracted a lot of attention in recent years due to their wide range of actions and mostly unexplored functions. For instance, lncRNAs have the potential to act as competitive endogenous RNAs, serve as protein scaffolds, assist in histone modification, or function as decoys to repress transcription (11–13). Recent research has found that lncRNAs are dysregulated in a variety of cancers, and lncRNA-based prognostic markers have been proposed for tumor categorization and patient survival prediction (14–17). Even though the accumulating studies underscored its key roles in gene regulation and subsequent effect on adult cancer progression in the past two decades, the studies about lncRNAs in childhood cancer development and its potential therapeutic application are still deficient (**Figure 1**). In this review, we summarize current knowledge of function of lncRNAs on the progression of pediatric cancers and discuss the promising application opportunity for lncRNAs in childhood cancer treatment, chiefly focusing on childhood solid tumor.

## Neuroblastoma (NB)

Neuroblastoma (NB) is derived from neural crest cells of the sympathetic nervous system during fetal development (18). It is the most common extracranial solid tumor in childhood and accounts for ~15% of childhood cancer-related mortality (19). Although the 5‐year survival rate for NB patients with low- and intermediate-risk disease is favorable, the rate for high-risk patients is less than 50% (20). High-throughput studies have revealed various genetic alterations and dysfunctional pathways that drive the initiation and cause the progression and resistance to therapy of NB (21). Many of these altered molecules, including lncRNAs, are being investigated in order to gain a better understanding of the disease and to find novel therapeutics for NB patients.

### LncRNA acts as oncogene

Small nucleolar RNA host gene (SNHG) is the host genes of snoRNA, which have been found to be dysregulated in many kinds of tumors (22). Recent studies have shown that aberrant expression of SNHGs is closely associated with the malignant progression of NB and the most common mechanism by which SNHGs drive NB development is through acting as competing endogenous RNA (ceRNA). One example of such studies is about SNHG7, which has been demonstrated to be upregulated in NB tissues and to play a key role in NB development and chemo-resistance. In this regard, SNHG7 binds to two microRNAs, miR-323a-5p and miR-342-5p, resulting in the upregulation of cyclin D1 expression, causing the progression of NB (23). Additionally, SNHG7 sponges miR-329-3p to increase the expression of MYO10, which is an actin-based molecular motor and plays a role in microtubule cytoskeletons integration during autophagosome formation, leading to the resistance to cisplatin of NB cells (24). By acting as ceRNA, SNHG7 also has been reported to interact with miR-653-5p, and thus promoting STAT2 expression (25). Another good example of SNHG acting as a ceRNA to promote NB malignancy is SNHG16. Yu et al. found that SNHG16 facilitated NB progression and highly expressed SNHG16 was positively correlated with poor clinical outcomes (26). Interestingly, SNHG16 binds to miR-542-3p and upregulates the expression of ATG5, a crucial component in the autophagy pathway, promoting proliferation and migration ability of NB cells (27). In addition, a recent report has also shown that SNHG16 can regulate the MAPK signaling pathway through the miR-542-3p/HNF4α axis, thereby contributing to the malignant progression of NB (28). Consistent with above results, silencing of SNHG16 dramatically suppresses the progression of NB (29, 30). SNHG4 levels were shown to be higher in NB tissues than in normal tissues. SNHG4 promotes NB proliferation, migration, and invasion by sponging miR-377-3p which has been demonstrated as an oncogene in many types of cancers (31).

In addition to SNHGs, there are some lncRNAs that drive the progression of NB through the ceRNA mechanism as well. For instance, lncRNA NHEG1 competes with miR-665 to negatively regulate its activity, expelling the inhibition on HMGB1 expression and promoting the aggressive phenotype of NB cells (32). It is worth mentioning that NHEG1 interacts with and stabilizes protein partner DEAD-box helicase 5 (DDX5), resulting in transactivation of β-catenin, elevated NHEG1 levels, and altered expression of downstream genes (33). Similarly, DLX6-AS1 is dramatically upregulated in NB tissues compared with normal tissues. By targeting of miR-513c-5p, miR-506-3p and miR−10, the DLX6-AS1 protects NB cells from cell cycle arrest and apoptosis and causes the progression of NB *in vivo via* multiple signaling pathway (34–36). Non-coding RNA activated by DNA damage (NORAD) is a highly conserved lncRNA necessary for genome stability. By interaction with two RNA-binding proteins–PUM1 and PUM2, NORAD maintains genomic stability. Accumulating studies have shown that NORAD functions as a ceRNA that regulates the downstream mechanisms of various cancers by sponging microRNAs (miRNAs), including NB. NORAD accelerates the progression and doxorubicin resistance of NB through up-regulating HDAC8 *via* sponging miR-144-3p (37). Various other lncRNAs, such as XIST, DUXAP8 and LINC01410, also have been shown to drive the progression of NB by ceRNA mechanism (38–40).

The ceRNA is not the only mechanism for lncRNA-mediated regulation, and lncRNA may interact with DNA, RNA, or protein to impact gene expression. LINC01296 provides an example of how lncRNA regulates downstream target function by interacting with protein. Through directly interaction with RNA binding protein (RBP) NCL, LINC01296 and NCL form a complex that could bind to promoter of oncogene SOX11 and activate its transcription. The LINC01296-NCL-SOX11 complex plays an important role in NB tumorigenesis and may serve as a prognostic biomarker as well as an effective therapeutic target for NB (41, 42). LncRNA pancEts-1 promotes the growth, invasion, and metastasis of NB cells by directly binding to RBP hnRNPK, and thus facilitate its physical interaction with β-catenin. Whereas hnRNPK inhibits proteasome-mediated degradation of β-catenin, resulting in transcriptional alteration of target genes associated with NB progression (43). Hepatocyte nuclear factor 4 alpha (HNF4A) derived long noncoding RNA (HNF4A-AS1) promotes aerobic glycolysis and NB progression. HNF4A-AS1 binds to heterogeneous nuclear ribonucleoprotein U (hnRNPU) to promote its association with CCCTC-binding factor (CTCF), resulting in transactivation of CTCF and transcriptional alteration of HNF4A and other genes associated with tumor progression (44).

MYCN oncogene amplification is observed in 20%–30% NB patients and the overall survival for these patients remains at less than 50% (45). Although MYCN amplification is best-characterized as the genetic marker of risk in NB, our understanding of the precise mechanisms of MYCN amplification, evaluation and potential interventions remains limited (46). Recently, some studies have demonstrated the regulatory roles of lncRNAs for MYCN dysregulation in NB patients. RNA sequencing analysis suggests that lncNB1 is most overexpressed in MYCN-amplified NB cells compared to MYCN-non-amplified cells. Mechanistically, lncNB1 binds to the ribosomal protein RPL35 to enhance E2F1 protein synthesis, leading to transcription activation of the GTPase-activating protein DEPDC1B gene. DEPDC1B induces ERK protein phosphorylation and MYCN protein stabilization. Notably, knockdown of lncNB1 significantly suppresses the clonogenic ability of NB cells *in vitro* and *in vivo* (47). MYCN opposite strand (MYCNOS) is a gene located on the antisense strand to MYCN, and MYCNOS-01 is an alternatively spliced transcripts in NB. MYCNOS-01 positively regulates MYCN protein level and affects growth of MYCN-amplified NB cells. Conversely, MYCN also negatively regulates MYCNOS-01 transcription by recruiting to the transcription start site of MYCNOS-01 (48). LncRNA MIAT was shown to be an upstream regulator of MYCN, as interference with its expression reduces the mRNA level of MYCN in NB cells (49). These results may hint that, although directly targeting MYCN or LncRNA is more challenging, we may disrupt the interaction between lncRNA and MYCN to reduce MYCN levels.

### LncRNA acts as tumor suppressor

Increasing evidence indicates that lncRNAs play complex and precise regulatory roles in cancer initiation and progression by acting as oncogenes or tumor suppressors (50). For instance, lncRNA cancer susceptibility candidate (CASC7) plays a tumor-suppressive role in several malignancies (51, 52). In particular, Zhou and colleagues reported that the level of CASC7 is dramatically lower in NB tissues than in adjacent non-tumor tissues. Overexpression of CASC7 inhibits NB cell proliferation by miR-10a mediated suppression of phosphatase and tensin (PTEN) homolog mRNA expression pathway (53). GWAS analysis and *in vitro* assays have demonstrated the tumor suppressor role of CASC15 in NB, but the underlying mechanism has not to be clarified (54). Later, results from a collaborative study showed that a pair of sense/antisense lncRNA encoded by CASC15 and NBAT1 promote differentiation through their regulatory interactions with key cancer-associated genes SOX9 and USP36. In detail, CASC15 and NBAT1 form a complex and then interact with USP36, leading to the nucleolar localization of USP36. Subsequently, USP36 is able to stabilize the CHD7 *via* its deubiquitinase activity, resulting in the activation of SOX9 and other oncogenes (55). Similarly, the lncRNA maternally expressed gene 3 (MEG3) also has been reported as tumor-suppressor in many types of cancers. In NB, downregulation of MEG3 facilitates NB malignant phenotype by stimulating ubiquitination degradation of EZH2. Of note, EZH2 in turn suppresses MEG3 expression by modulating H3K27me3 expression. Therefore, MEG3 and EZH2 may form a negative feedback loop to promote NB development (56). FOXD3-AS1 is downregulated in NB tissues and cell lines, and ectopic expression of FOXD3-AS1 stimulates neuronal differentiation and decreases the aggressiveness of NB cells *in vitro* and *in vivo*. Mechanistically, FOXD3-AS1 interacts with poly (ADP-ribose) polymerase 1 (PARP1) in nucleus to prevent poly (ADP-ribosyl)ation and activation of CTCF, resulting in decreasing of downstream tumor-suppressive genes (57).

### LncRNA acts as a predictive biomarker

Single-nucleotide polymorphisms (SNPs) in the genome cause changes in the structure and function of lncRNA. Accumulating evidence has indicated SNPs on lncRNAs may have a lot of promise as biomarkers. As mentioned above, CASC15 is well-studied as a tumor suppressor in NB. While rs9295534, a NB susceptibility locus located in the upstream enhancer of CASC15-S, has recently been shown as a risk allele at NB susceptibility loci (58). The genetic polymorphism of LINC00673 is believed to affect the susceptibility of a population to the corresponding childhood cancer types, including NB (59–61). Lnc-LAMC2–1:1 rs2147578 C > G polymorphism may contribute to NB susceptibility (62)

## Osteosarcoma (OS)

Osteosarcoma is a type of bone cancer that begins in the cells that form bones. It is the most common type of primary malignant bone tumor among adolescent patients (63). Notably, nearly 10-20% of patients have measurable metastasis prior to onset, with the lungs being the most common site. The presence of metastases is a clear indicator of a poor overall prognosis. In fact, the prognosis of such patients is almost entirely determined by metastasis and drug resistance, particularly the presence of lung metastases (64, 65). Deep sequencing with samples from primary OS, pulmonary metastatic OS, and normal controls revealed that the landscape of lncRNA is dynamically regulated in progression of OS (66).

### LncRNA acts as oncogene

LncRNA RBM5-AS1 is a nuclear-retained transcript that selectively interacts with β-catenin (67). RBM5-AS1 is overexpressed in the OS tissues and cell lines, and the upregulated RBM5-AS1 promotes OS cell proliferation, migration, and invasion *in vitro* and tumor growth *in vivo* by (68). The lncRNA C5orf66 antisense 1(C5orf66-AS1), a recently discovered lncRNA, has been reported to be associated with the pathogenesis of OS as well as other types of tumors, including cervical cancer, oral squamous cell carcinoma, liver cancer. C5orf66-AS1 epigenetically downregulates MMP3 by acting as a ceRNA for miR-149-5p that represses MMP3 expression, thus promoting proliferation and invasion of OS cells (69). MALAT1 (metastasis associated lung adenocarcinoma transcript-1) is a 7.5-kb long lncRNA that was discovered to be overexpressed in non-small cell lung cancers. MALAT1 is also found to be overexpressed in OS and other cancers including breast cancer, prostate cancer, and ovarian cancer (70). Of interest, bone marrow mesenchymal stem cells-derived extracellular vesicles can be absorbed by OS, and MALAT1 delivered by extracellular vesicles (EVs) derived from bone marrow mesenchymal stem cells (BMSC-EVs) affects the viability and morphology of OS cells (71). Similarly, lncRNA PVT1 encapsulated in BMSC-derived exosomes promotes OS growth and metastasis by stabilizing ERG and sponging miR-183-5p (72). LncRNA EPIC1 has been implicated in human osteoblasts. Recent reports have shed light on its function in OS development by mediating the ubiquitylation of transcription factor MEF2D (73). Various other lncRNAs including SNHG16, LINC00266-1, NR_136400, and Sox2OT-V7 also promote the progression and/or chemoresistance of OS by ceRNA mechanism (74–79).

### LncRNA acts as a predictive biomarker

The long non-coding RNA ANRIL, antisense to the CDKN2B locus, controls cell proliferation and senescence *via* regulating its neighboring tumor suppressors CDKN2A/B by epigenetic mechanisms (80). Polymorphisms at the ANRIL gene are not only linked to risk for many different cancers, but also to atherosclerotic cardiovascular disease, bone mass, obesity and type 2 diabetes. It has been previously characterized as a predict biomarker for many types of adult cancers (81). Recently, ANRIL has been identified to be associated with increased resistance to two standard-of-care chemotherapeutic agents, cisplatin and doxorubicin, in in both HOS and U2OS cell lines of OS. Analysis of the Therapeutically Applicable Research To Generate Effective Treatments (TARGET) OS patient cohort showed that higher ANRIL expression is significantly associated with poor outcomes and metastases (82). Similarly, high level of HULC is also link to poor prognosis of OS patients (83, 84). Yu and colleagues interrogated the OS dataset from TARGET (84). Among a total of 97 samples that have RNA-seq results, they found 24 metastatic samples and 73 non-metastatic samples; and then identified RP11-128N14.5, RP11-231|13.2, RP5-894D12.4, LAMA5-AS1 and RP11-346L1.2 as metastasis-related lncRNAs that can be used as potential prognostic indicator for OS.

## Wilms Tumor (WT)

Renal malignancies account for 7% of all childhood cancers and include multiple distinct subtypes that differ greatly in appearance and prognosis. Wilms tumor (WT), also known as nephroblastoma, accounting for 90% of cases (85, 86). However, in addition to genetic susceptibility, external causative factors for WT have not been identified.

### LncRNA acts as oncogene

LncRNAs implicated in WT development include LINC00473, SOX21-AS1, LINC00667, SNHG6, HOXA11-AS, MYLK-AS1 and XIST (87–93). For example, by recruiting forkhead box P2, HOXA11-AS upregulates cyclin D2 to prevent apoptosis and accelerate cell cycle progression in WT (91). LncRNA MYLK-AS1 has been reported to be involved in progression and chemoresistance of many digestive system tumors, such as gastric cancer, gallbladder carcinoma and hepatocellular carcinoma (94–96). MYLK-AS1 recruited TCF7L2 to regulate CCNE1 in mediation on cell proliferation and cell cycle distribution of WT (92). Zhu and colleagues discovered that LINC00473 was up-regulated in WT tissues and was associated with a higher clinical stage and unfavorable WT histology. Mechanistically, LINC00473 activates the IKK signal pathway by sponging miR-195, resulting in the progression of WT (87). XIST (X-inactive specific transcript) induces X-inactivation and is aberrantly expressed in malignant tumors, including WT, colorectal cancer, liver cancer, and gastric cancer (97). In WT, XIST promotes expression of YAP by interacting with miR-194-5p (93).

### LncRNA acts as a predictive biomarker

A number of lncRNAs are implicated as biomarkers for WT. Analysis of nephroblastoma data from the TARGET database found that eight lncRNAs (AC079310.1, MYCNOS, LINC00271, AL445228.3, Z84485.1, AC091180.5, AP002518.2, and AC007879.3) were significantly overexpressed in WT tissue, and were linked with prognosis in WT (98). As a new form of programmed cell death, ferroptosis has gained enormous interest in cancer research communities. Accumulating evidence has demonstrated that dysregulated ferroptosis is a significant cause of tumor initiation and development (99). Liu and colleagues identified 12 ferroptosis-related lncRNAs whose expression correlated with the prognosis of WT patients and could be used as prognostic predictor markers by interrogating the TARGET WT dataset (100).

## Rhabdomyosarcoma (RMS)

Rhabdomyosarcoma (RMS) is the most common soft tissue sarcoma in children arising from primitive embryonal mesenchyme (101). RMS is currently categorized into four histological subtypes, with embryonal (ERMS) and alveolar (ARMS) being the most prevalent. ARMS is often characterized by the presence of a fusion oncoprotein, namely paired box 3-forkhead Box O1 (PAX3-FOXO1) or PAX7-FOXO1 and it is clinically more aggressive than ERMS (102). PAX3-FOXO1 drives the transcription of the oncogene MYCN, which helps cells grow in ARMS. The lncRNA MYCNOS-01 derived from the antisense strand of MYCN was shown to upregulate MYCN protein levels and promote the growth of MYCN-amplified RMS and NB (48). Loss of imprinting (LOI) by DNA hypermethylation at the differentially methylated region (DMR) for the IGF2-H19 locus is commonly observed in RMS cells and results in downregulation of H19. H19 has dual roles in cancer progression steps in which it can promote tumorigenesis, it can also operate as a tumor suppressive lncRNA *via* a distinct mode of action (103). It has been reported that the expression of the H19 gene is significantly downregulated in ERMS compared to normal muscle tissue. As a precursor, H19 generates miR-657, which suppresses the growth of RMS (104).

## Retinoblastoma (RB)

Retinoblastoma (RB) is the most common primary intraocular malignancy of childhood. It usually initiates due to the biallelic mutation of the retinoblastoma gene (RB1) in a single susceptible developing retinal cell (105, 106). MIR17HG, the host gene of miR-17-92 cluster, plays a significant role in regulating many types of tumor progression, including RB, lung cancer, colorectal cancer and glioma (107–110). Especially it enhances RB cell proliferation, migration, and invasion by elevating HIF-1expression through sponging miR-155-5p (110). LncRNA HOTAIR is elevated in RB cells relative to that in normal retinal cells. It upregulates Ribonucleotide Reductase Regulatory Subunit M2 (RRM2) by competitively binding to miR-20b-5p and activates PI3K/AKT pathway, thereby promoting proliferation and repressing apoptosis of RB cells (111). In addition, a number of lncRNAs are implicated in RB progression by acting as ceRNA, including HOTAIR, UCA1, CCAT1, MALAT1, XIST, NEAT1 and CASC9 (112–117). However, some other lncRNAs were found to be abnormally expressed in RB and involved in its pathological process but the function is not clear yet.

## Hepatoblastoma (HB)

Hepatoblastoma, which is a rare malignant embryonal tumor with divergent patterns of differentiation, almost exclusive occurs in childhood. LncRNAs implicated in HB, such as MIR205HG, NBR2, CRNDE and PVT1, that have been demonstrated to be upregulated in HB. MIR205HG competitively binds to miR-514a-5p and targets mitogen-activated protein kinase 9 (MAPK9) to stimulate MAPK signaling pathway. Meanwhile, MIR205HG also serves as a sponge for miR-205-5p to activate the PI3K/AKT signaling pathway. Taken together, MIR205HG accelerates cell proliferation, migration and invasion in HB through MAPK and PI3K/AKT signaling pathways (118). The expression of NBR2 is significantly increased within HB samples; moreover, under glucose starvation, NBR2 expression is significantly upregulated. NBR2 counteracts miR-22-mediated repression on TCF7 *via* acting as a ceRNA, resulting in not only the increasing of HB cell viability, invasion, and migration under normal culture condition but also the resistance of cell apoptosis under glucose starvation (119). Finally, CRNDE is upregulated in HB tissues and is able to promote HB cell angiogenesis by targeting the miR-203/VEGFA axis (120). Although, PVT1 has been identified as oncogene in both adult and pediatric malignancies. However, its precise mechanism of action in hepatoblastoma remains unknown.

## Medulloblastoma (MB)

Medulloblastoma (MB) is the most common central nervous system (CNS) embryonal tumor. MB is primarily classified into four subgroups based on molecular and clinical characteristics as (1) WNT (2) Sonic-hedgehog (SHH) (3) Group 3 (4) Group 4. MB subgroups share genomic and mRNA profiles and require multiple molecular markers for differentiation from each other. Even though the overall cure rate is approximately 70%, patients with high-risk diseases continue to have poor outcomes and long-term morbidity (121). There is a pressing need to identify the underlying molecular pathways in these subgroups in order to promote precision medicine-based therapies, improve quality of life, and expand our overall understanding of MB (122). By searching Gene Expression Omnibus (GEO) database for MB related microarray datasets, Kesherwani and colleagues determined the prognostic relevance of lncRNAs and the distinct lncRNAs associated with each MB subgroup (123). Through a joint analysis of the RNA-seq data from the European Genome-Phenome Archive (EGA) and two studies of MB from GEO, the research team from Johns Hopkins University identified 17 putative candidate lncRNAs that could be used as progression or as diagnostic and prognostic biomarkers (124). A recent investigation revealed exceptional activity for three MYC-dependent lncRNAs, lncMB1, lncMB2, and lncMB3, in group 3 MB landscape, and identified their target gene(s), which may aid future research focused on constructing novel regulatory circuits that are altered in MB (125). Nevertheless, very few lncRNAs have been investigated for their precise functions and mechanisms in MB or its subtypes at the present. NKX2–2AS modulates SHH-induced MB formation *in vitro* by functioning as miRNA sponge for miR-103 and miR-107, thereby de-repressing their tumor suppressive targets BTG2 and LATS1 and limiting tumor cell proliferation and migration (126). HOTAIR promotes MB development, migration, and invasion sponging miR-1/miR-206, and thus elevating their target gene YY1 expression (127). CCAT1 has been demonstrated to promote tumor growth and metastasis by activating the MAPK pathway in WNT and group 3 MBs (128). Other lncRNAs, including CRNDE, UCA1, lnc-HLX-2-7 and LOXL1-AS1 are also reported as an oncogenic lncRNA in MB. However the mode of action of these lncRNAs remains unclear (129–132).

## Conclusion and perspective

**Figure 2:**
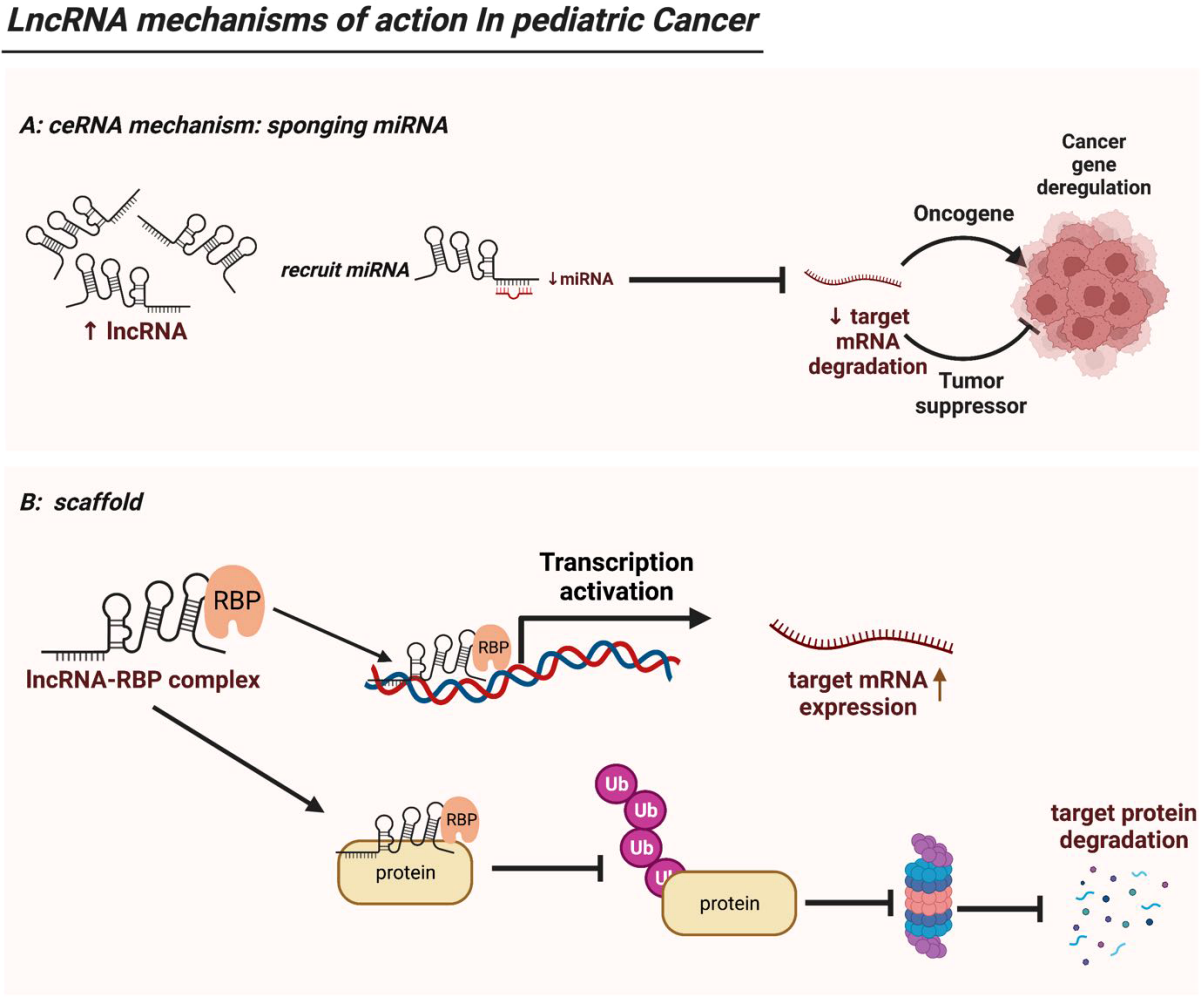
LncRNA mechanisms of action in pediatric cancer. **(A)** LncRNAs serve as ceRNAs by competitively occupying miRNAs’ shared binding sequences, thus sequestering the miRNAs and altering the expression of their downstream target genes. **(B)** LncRNAs act as modular scaffolds. By interacting with RBP, lncRNA-RBP complex can bind to promoter region of a target gene thus driving its gene expression. In addition, the lncRNA-RBP complex can mediate or sequester ubiquitination of target protein, thereby regulating downstream protein expression. .

There is an urgent need to identify novel therapies for childhood cancers. LncRNAs have caught a great deal of attention over the past decades because their wide range of expression patterns in various types of cancer and their tumor specificity provide a new basis for developing diagnostics and therapeutics in a variety of cancer. However, lncRNA research in pediatric cancer has been carried out very slowly due to a number of obstacles. First, despite the enormous number of lncRNAs identified so far and their dysregulation reported in diverse malignancies, systematic identification and characterization of childhood cancer-related lncRNAs is at its infancy **Table 1**. Second, quality clinical specimens or relevant databases for pediatric cancer are difficult to obtain. Third, overall research effect on the function and application of lncRNAs in pediatric and adolescent malignancies is less than in adult tumors **Figure 2**. However, pediatric cancers have its own unique features which may advantage over the adult cancers. For instance, most of childhood tumors are derived from mesoderm whereas adult tumors are mostly epithelium-derived, which may suggest that lncRNA expression could be very different. Some lncRNAs could be childhood cancer specific. In addition, early diagnosis of pediatric cancer, especially for early aged children, which present a more urgent need for biomarkers for this type of cancers, and thus this provides a great opportunity for researchers in their careers. In summary, elucidation of the function of lncRNAs and association with diverse subtypes of childhood cancer, and development of novel lncRNA-based approaches for diagnostics and targeted therapy hold considerable potential, despite the challenges ahead.

**Table 1:** Features different types of lncRNAs and their roles in pediatric cancer.

Type of Pediatric Cancer	Long non-coding RNA			Mode of function	Role
**Neuroblastoma (NB)**	SNHG7, SNHG16, SNHG4			**ceRNA mechanism**	**Oncogene**
	NHEG1				
	DLX6-AS1				
	NORAD				
	XIST				
	DUXAP8				
	LINC01410				
	LINC01296			**serving as scaffold**	
	PancEts-1				
	HNF4A-AS1				
	LncNB1			**regulator for MYCN dysregulation**	
	MYCNOS-01				
	CASC7			**ceRNA mechanism**	**Tumor suppressor**
	CASC15,NBAT1			**serving as scaffold**	
	MEG3				
	FOXD3-AS1				
	CASC15-S (rs9295534)			**NA**	**Biomarker**
	Lnc-LAMC2-1:1(rs2147578)				
	LINC00673				
**Osteosarcoma (OS)**	C5orf66-AS1			**ceRNA mechanism**	**Oncogene**
	PVT1				
	SNHG16				
	NR_136400				
	LINC00266-1				
	Sox2OT-V7				
	MALAT1				
	EPIC1			**serving as scaffold**	
	RBM5-AS1			**not determined**	
	PARTICLE				
	ANRIL			**NA**	**Biomarker**
	HULC				
	RP11-128N14.5				
	RP11-231|13.2				
	RP5-894D12.4				
	LAMA5-AS1				
	RP11-346L1.2				
**Wilms Tumor (WT)**	LINC00473			**ceRNA mechanism**	**Oncogene**
	XIST				
	SNHG6				
	LINC00667				
	MYLK-AS1	**serving as scaffold**			
	SOX21-AS1			**not determined**	
	AC079310.1			**NA**	**Biomarker**
	MYCNOS				
	LINC00271				
	AL445228.3				
	Z84485.1				
	AC091180.5				
	AP002518.2				
	AC007879.3				
**Rhabdomyosarcoma (RMS)**	MYCNOS-01			**not determined**	**Oncogene**
	H19			**ceRNA mechanism**	**Tumor suppressor**
**Retinoblastoma (RB)**	MIR17HG			**ceRNA mechanism**	**Oncogene**
	HOTAIR				
	UCA1				
	CCAT1				
	MALAT1				
	XIST				
	NEAT1				
	CASC9				
**Hepatoblastoma (HB)**	MIR205HG			**ceRNA mechanism**	**Oncogene**
	NBR2				
	CRNDE				
	PVT1			**not determined**	
**Medulloblastoma(MB)**	NKX2–2AS			**ceRNA mechanism**	**Oncogene**
	HOTAIR				

## Author contributions

FL and Q-WX researched and drafted the article. J-HW and W-XP supervised the content. All authors contributed to the article and approved the submitted version.
